# Arthroscopic 3‐Point Suture‐Bridge Fixation for Posterior Cruciate Ligament Tibial Avulsion Fractures via Triple Bone Tunnels

**DOI:** 10.1002/atn2.70382

**Published:** 2026-11-05

**Authors:** Wuping Yao, Pengchao Liu, Zheyuan Li, Xiaoming Xin, Yuji Li, Houyi Jia, Zhi Liu

**Affiliations:** ^1^ Department of Orthopedics Gansu Provincial Hospital of Traditional Chinese Medicine Lanzhou China; ^2^ Gansu University of Chinese Medicine Lanzhou China

## Abstract

Posterior cruciate ligament tibial avulsion fractures are a distinct clinical entity. Although the optimal fixation strategy for these fractures remains debated, arthroscopic fixation emerges as a preferred surgical approach owing to its minimally invasive nature, superior intra‐articular visualization, and effectiveness in restoring ligamentous tension and joint stability. In this technical note, we use a 3‐point fixation technique involving 3 small‐diameter tibial bone tunnels for the management of a posterior cruciate ligament tibial avulsion fracture. This approach facilitates precise fracture reduction and significantly enhances postoperative knee joint stability.

## Graphical abstract

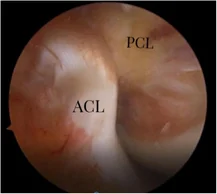

**VIDEO 1:** Surgical technique of arthroscopic 3‐point suture‐bridge fixation for posterior cruciate ligament (PCL) tibial avulsion fractures of the right knee. The patient is placed in the supine position with the knee flexed at 90°. Diagnostic arthroscopy is first performed through the standard anteromedial and anterolateral portals to assess the intra‐articular structures. The posteromedial and posterolateral portals are then established under arthroscopic visualization to expose the avulsion fragment at the tibial attachment of the PCL. Under arthroscopic visualization through the posterolateral portal, an anterior cruciate ligament tibial guide is used to create the proximal bone tunnel, followed by the creation of the distal medial and distal lateral bone tunnels. Two high‐strength sutures with a button plate are passed through the proximal bone tunnel from anterior to posterior, exiting adjacent to the avulsion fragment and traversing the center of the PCL. The sutures are adjusted to encircle and sandwich the avulsion fragment. The free ends of the sutures are then passed through the distal medial and distal lateral bone tunnels, respectively. The sutures are tensioned to achieve anatomical reduction of the avulsion fragment, tied over the anterior tibial border, and further secured with an external row anchor on the anterior tibial cortex. Final arthroscopic assessment from the anterolateral portal confirms restoration of PCL tension and stable fixation of the fragment. Video content can be viewed at https://doi.org/10.1002/atn2.70382.

At present, surgical intervention remains the gold standard treatment for displaced tibial avulsion fractures of the posterior cruciate ligament (PCL).^1^ Both open reduction and internal fixation and arthroscopic techniques are effective in managing PCL tibial avulsion fractures.^2^, ^3^, ^4^ Nonetheless, arthroscopic management gains popularity because it optimizes postoperative functional recovery, minimizes surgical trauma, and reduces infection risk.^4^ Various fixation modalities are described, including suture fixation, cannulated screw fixation, and cortical button suspension.^5^ Recently, several novel arthroscopic techniques have been reported. Wu et al.^6^ adapted the suture‐bridge technique from rotator cuff repair to PCL tibial avulsion fractures, using 2 high‐strength FiberWire sutures (Arthrex, Naples, FL) passed through 2 tibial tunnels and secured with an external row anchor. Capella et al.^7^ described an all‐inside double‐tunnel pullout technique with high‐strength suture tape augmentation via a trans‐septal approach, incorporating an InternalBrace (Arthrex, Naples, FL) ligament augmentation system to enable early rehabilitation. Thonse et al.^8^ introduced a crisscross fixation technique using 2 FiberTape sutures (Arthrex, Naples, FL) crossed over the avulsed fragment and fixed anteriorly with a suture button. In this technical note, we describe an arthroscopic 3‐point fixation technique with triple bone tunnels to treat PCL tibial avulsion fractures. This technique ensures effective reduction and stable fixation of the fracture fragment and facilitates the anatomical restoration of PCL tension.

## SURGICAL TECHNIQUE

### Patient Position, Surgical Approach, and Arthroscopy

The patient is placed in the supine position with knees flexed at 90°. Using standard anterior medial and anterior lateral arthroscopic approaches, the articular cartilage, medial meniscus, lateral meniscus, anterior cruciate ligament (ACL), and PCL within the joint cavity are examined. ACL is lax because of posterior tibial displacement (Figure 1). Under arthroscopic guidance, posterior medial and posterior lateral approaches are established to expose the avulsion fracture fragment at the PCL insertion site, followed by debridement of the coagulated blood clots and fibrous scar tissue at the fracture margins.

**FIGURE 1:**
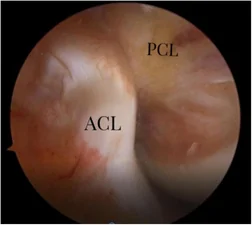
Arthroscopic view of the right knee joint from the anterolateral portal with the patient in the supine position and the knee flexed at 90°. Significant laxity of the ACL resulting from the posterior sagging of the tibia because of the PCL avulsion fracture can be seen. (ACL, anterior cruciate ligament; PCL, posterior cruciate ligament.)

### Establishment of Bone Tunnel

An ACL tibial locator (Naton, Beijing, China) is used to position the tibial end of the PCL anteriorly near the cartilage margin (Figure 2A). A 2‐mm bone tunnel (proximal bone tunnel) is drilled from anterior to posterior, and a polydioxanone (PDS) suture is introduced using a lumbar puncture needle (Figure 2B). Subsequently, the ACL tibial locator is used to drill two 2‐mm bone tunnels (distal medial and distal lateral bone tunnels) at the distal end of the fracture, both slightly medial and lateral (Figure 2C). The PDS suture is then introduced using the same method (Figure 2D).

**FIGURE 2:**
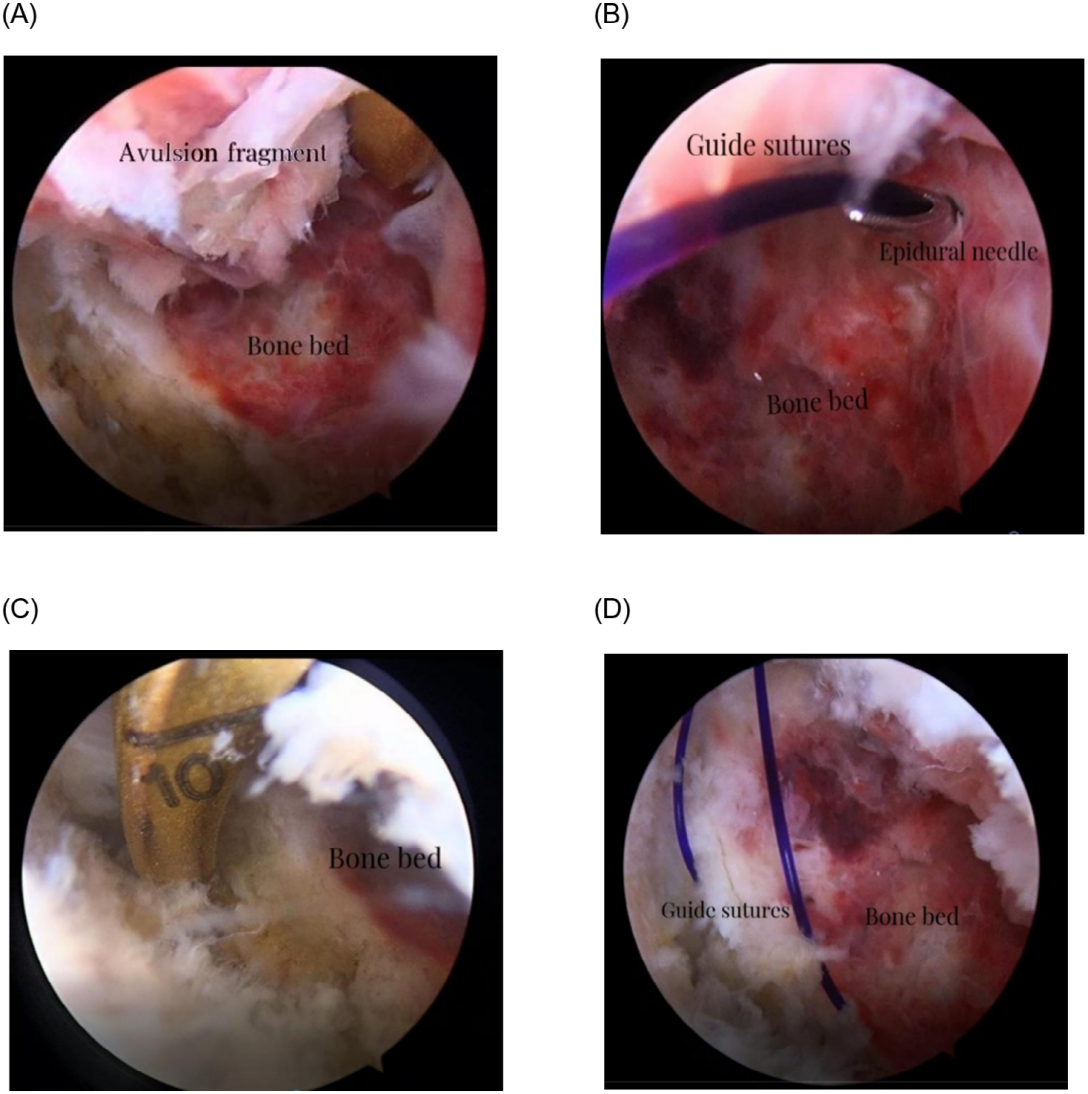
Establishment of the bone tunnels for the right knee. The patient is placed in the supine position with the knee flexed at 90°. Arthroscopic views are obtained through the posterolateral portal. (A) Intraoperative visualization of the PCL tibial insertion site. An ACL tibial guide is positioned at the anterior aspect of the PCL footprint, adjacent to the articular cartilage margin, to prepare the proximal bone tunnel. (B) Creation of the proximal bone tunnel. A 2‐mm bone tunnel is drilled, and a PDS suture is introduced as a shuttle through a spinal needle. (C,D) Preparation of the distal bone tunnels. Two 2‐mm bone tunnels are drilled at the distal part of the fracture site, (C) 1 medial and 1 lateral, followed by the (D) introduction of PDS shuttles. (ACL, anterior cruciate ligament; PCL, posterior cruciate ligament; PDS, polydioxanone.)

### Reduction and Fixation of Fracture Fragments

The proximal bone tunnel PDS line is introduced with 2 high‐strength sutures (Blue, Arthrex, Pendleton, SC; Blue and White, Naton, Beijing, China) equipped with a button plate (Arthrex, Pendleton, SC) (Figure 3A). The titanium plate is placed laterally on the anterior border of the tibia. The distal ends of the high‐strength sutures are introduced from the posterior compartment into the anterior compartment, and the PDS line is secured to the bone mass via an epidural needle, passing through the center of the PCL before the high‐strength sutures are introduced into the posterior compartment (Figure 3B and 3C). The tail wires are then passed through both distal bone tunnels, and the fracture fragments are reduced (Figure 3D). After tying and securing the anterior border of the tibia, all tail wires are anchored with a single external row anchor (Arthrex, Pendleton, SC) (Figure 3E). The tension in the PCL, reduction of the fracture fragments, and recovery of ACL tension are reassessed (Figure 3F). Figure 3G shows the postoperative image of the patient's knee. The surgical schema is shown in Figure 4. The surgical procedure is shown in the accompanying video (Video 1).

**FIGURE 3:**
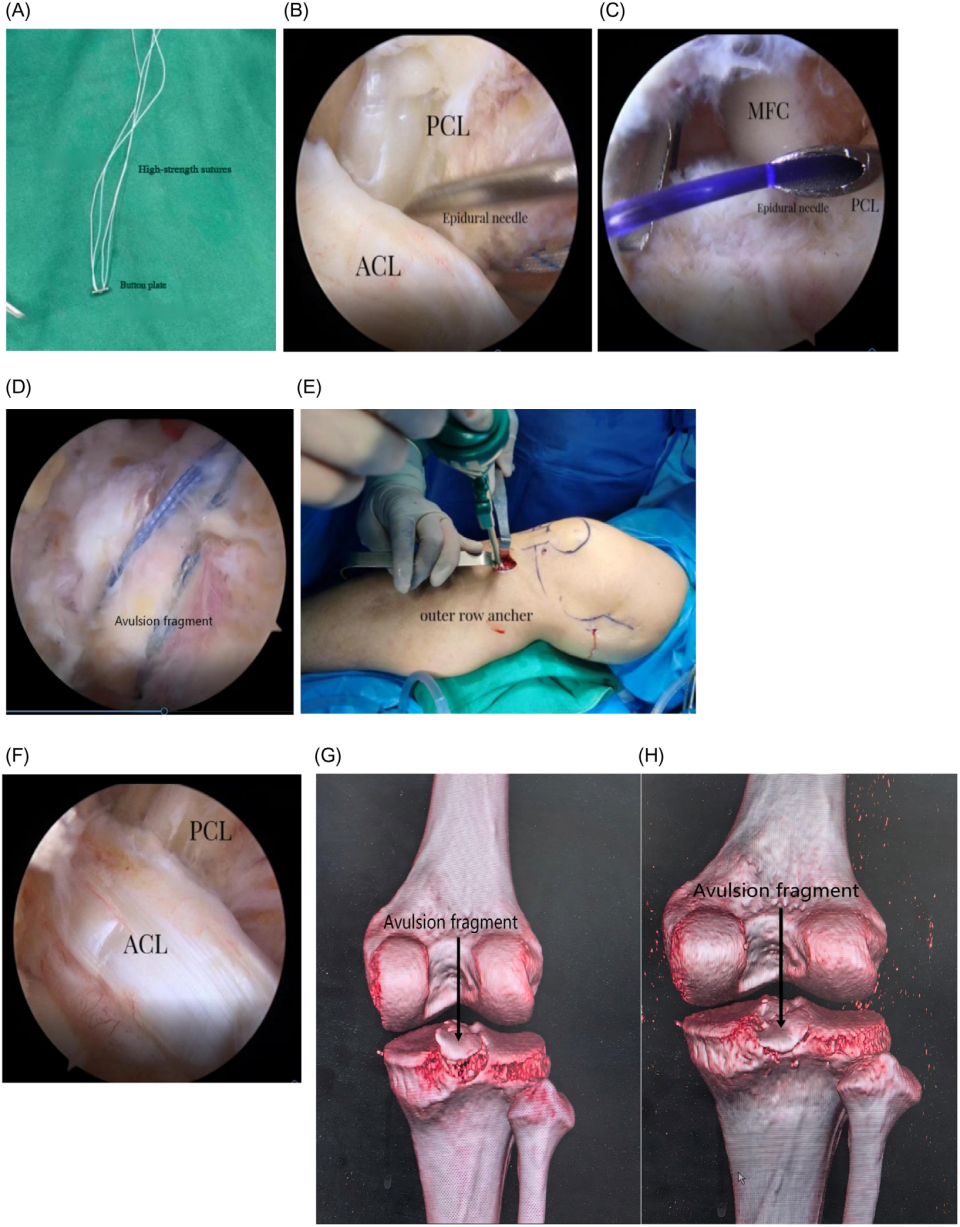
Reduction and fixation of the PCL tibial avulsion fracture in the right knee. The patient is placed in the supine position with the knee flexed at 90°. (A) Suture management. Two high‐strength sutures attached to a button plate are pulled through the proximal bone tunnel. The plate is secured against the anterior tibial cortex. (B,C) Suture retrieval. (B) An epidural needle is used to pierce the PCL substance immediately proximal to the avulsion fragment under arthroscopic visualization from the anterolateral portal. (C) The sutures are then retrieved through the PCL and into the posterior compartment, viewed from the posterolateral portal. (D) Fracture reduction. The high‐strength sutures are passed through the distal medial and distal lateral bone tunnels, and the avulsion fragment is anatomically reduced to its original footprint, visualized through the posterolateral portal. (E) Final fixation. After tying the sutures over the anterior tibial bone bridge, all suture ends are further secured using an additional external row anchor to maximize construct stability. (F) Final arthroscopic assessment from the anterolateral portal. Proper tensioning of the PCL is confirmed, the fracture fragment is stably fixed, and the previously noted ACL laxity is successfully restored. (G,H) Radiographic evaluation. (G) A preoperative CT scan shows a displaced PCL tibial avulsion fracture, and (H) the postoperative CT scan confirms anatomical reduction and stable internal fixation of the fragment. (ACL, anterior cruciate ligament; CT, computed tomography; MFC, medial femoral condyle; PCL, posterior cruciate ligament.)

**FIGURE 4:**
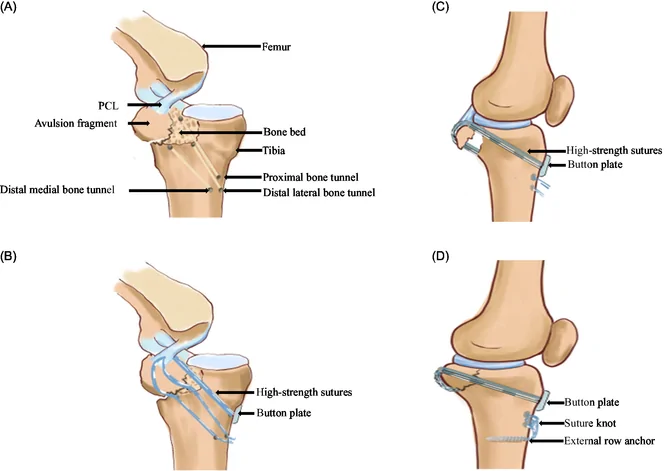
Schematic illustrations of the 3‐point suture‐bridge fixation technique for PCL tibial avulsion fractures of the right knee. The patient is placed in the supine position with the knee flexed at 90°. After the intra‐articular structures are examined through standard anteromedial and anterolateral arthroscopic portals, the posteromedial and posterolateral portals are established under arthroscopic visualization to expose the avulsion fragment at the tibial attachment of the PCL. (A) Posterior coronal view showing the avulsion fragment displaced from its bone bed at the tibial attachment of the PCL, with loss of PCL tension. The proximal bone tunnel and the distal medial and distal lateral bone tunnels are depicted on the tibia. (B) An oblique sagittal view shows 2 high‐strength sutures with a button plate passed through the proximal bone tunnel from anterior to posterior, exiting adjacent to the avulsion fragment and traversing the center of the PCL. The sutures are then adjusted to encircle and sandwich the avulsion fragment. The free ends of the sutures are subsequently passed through the distal medial and distal lateral bone tunnels, respectively, from posterior to anterior. (C) Sagittal view showing the same step as (B), with the 2 high‐strength sutures passed through the proximal bone tunnel, adjusted to encircle the avulsion fragment, and their free ends passed through the distal medial and distal lateral bone tunnels. (D) Sagittal view after final fixation. The high‐strength sutures are tensioned to achieve anatomical reduction of the avulsion fragment. The sutures are then tied over the anterior tibial border, and all suture tails are further secured with an external row anchor on the anterior tibial cortex, restoring the tension of the PCL. (PCL, posterior cruciate ligament.)

## DISCUSSION

The PCL is a critical stabilizing structure of the knee joint. Fractures involving the tibial insertion of the PCL result in a complete loss of ligamentous function, leading to significant joint instability.^4^ Current evidence suggests that both open reduction and arthroscopic repair yield comparable clinical outcomes.^2^, ^3^ However, open surgery is often hindered by restricted visualization and carries risks of neurovascular injury, wound dehiscence, joint infection, and arthrofibrosis. Furthermore, they also require a secondary operation to remove the hardware. In contrast, arthroscopic repair emerges as the mainstream approach because of its superior visualization and minimally invasive nature.

Common arthroscopic fixation modalities include suture fixation, cannulated screw fixation, and cortical button suspension.^9^, ^10^, ^11^ Among the recently described techniques, the suture‐bridge technique provides uniform suture distribution over the fracture fragment and reduces the risk of suture displacement by using an external row anchor for distal fixation.^6^ The double‐tunnel pullout technique with suture tape augmentation, performed through a trans‐septal approach, offers enhanced fixation strength and allows early functional recovery through the addition of an InternalBrace ligament augmentation system.^7^ The crisscross technique, using 2 FiberTape sutures crossed over the avulsed fragment, provides multidirectional compression and secure fixation with a suture button on the anterior tibial cortex.^8^ Although simple dual‐tunnel suture fixation may result in suboptimal fracture reduction and delayed union,^5^ triple‐tunnel suture fixation provides substantially greater construct strength than dual‐tunnel techniques, achieving stability comparable to that of screw or cortical button fixation.^12^ Moreover, the triple‐tunnel technique is shown to enhance fracture reduction substantially and mitigate posterior instability.^13^, ^14^ In the present technical note, we use a 3‐point fixation technique with triple bone tunnels passing through the PCL, which ensures effective reduction while simultaneously enhancing fixation strength.

Several critical technical considerations should be noted during the procedure. First, thorough exposure of the fracture fragment is essential, and the proximal tunnel should be created using an ACL tibial guide positioned slightly posterior to the articular cartilage margin. Additionally, sutures should be passed through the PCL substance in proximity to the bone‐ligament interface to minimize the “cheese‐wiring” effect on the ligament. Furthermore, anatomical reduction of the fracture fragment must be achieved before final suture tensioning and fixation (Table 1). Finally, all sutures must be meticulously positioned over the surface of the avulsion fragment to prevent fragment tilting during tensioning.

**TABLE 1:** Pearls and Pitfalls

**Technical Pearls**	**Technical Pitfalls**
Thorough debridement of the fracture site to ensure clear visualization of the proximal tunnel landmark.	Inadequate exposure of the fracture site, particularly in cases of large avulsion fragments, may lead to inaccurate identification and malpositioning of the proximal tunnel.
Use of 2.0‐mm bone tunnels throughout the procedure, ensuring adequate exposure of the anterior tibial cortex for precise tunnel localization.	Premature tensioning of sutures before achieving anatomical reduction may result in asymmetric tilting or malalignment of the avulsion fragment.
Suture shuttling through the bone tunnels is accomplished using an epidural needle as a carrier for PDS sutures.	
Strategic placement of both outer row tunnels distal to the fracture site.	
When passing through the PCL via the proximal tunnel, the sutures should be positioned in close proximity to the fracture site to minimize the “cheese‐wiring” effect on the ligament.	
Suture management and tensioning: sutures must be precisely centered over the avulsion fragment and tensioned only while maintaining anatomical fracture reduction.	
The trailing ends of the sutures are finally secured using a single external row anchor.	

PCL, posterior cruciate ligament; PDS, polydioxanone.

Despite its strengths, the present study has certain limitations. First, for large fracture fragments characterized solely by posterior upward tilting, the exposure and creation of the proximal tunnel may be technically challenging. Second, the requirement for frequent arthroscopic switching between the anterior and posterior compartments increases the technical complexity of the procedure, as summarized in Table 2 along with the strengths of this technique.

**TABLE 2:** Advantages and Limitations of the Technique

**Advantages**	**Limitations**
Anatomical reduction: the triple‐tunnel technique facilitates more precise reduction and multipoint fixation of the fragment.	Technical challenges in specific fracture patterns: in cases involving large avulsion fragments with isolated posterior upward tilting, achieving adequate exposure of the inner‐row tunnel site may be technically demanding.
Superior mechanical stability: it provides significantly higher biomechanical fixation strength at the fracture interface.	Increased technical complexity: frequent switching of the arthroscope between the anterior and posterior compartments increases the overall technical demand and may prolong the operative time.
Reinforced inner row: the combination of a suspension titanium plate and high‐strength sutures ensures robust inner‐row stability and initial construct strength.	

## DISCLOSURES

The authors (W.Y., P.L., Z.L., X.X., Y.L., H.J., Z.L.) declare the following financial interests/personal relationships which may be considered as potential competing interests: W.Y., P.L., Z.L., X.X., Y.L., H.J., and Z.L. receive support for the present manuscript (e.g., funding, provision of study materials, medical writing, and article processing charges): Exploring the Effects of Bushen Huoxue Decoction on Anterior Cruciate Ligament Based on the Theory of “Kidney Governing Bones” Clinical Study on Postoperative Bone Tunnel Expansion and Knee Joint Function Impact After Reconstruction Surgery (GZKZ‐2025‐11).

## FUNDING

This work was supported by the Liu Zhi Famous Traditional Chinese Medicine Practitioner Studio of Gansu Province (Grant No. Gancaishe (2025) No. 144).
